# Emphysematous Osteomyelitis of the Pubis Associated With Necrotizing Soft Tissue Infection of the Thigh

**DOI:** 10.5435/JAAOSGlobal-D-21-00088

**Published:** 2023-05-02

**Authors:** Tyler J. Moon, Jason Ina, Yazdan Raji, Anokha Padubidri, John Sontich

**Affiliations:** From the Department of Orthopedic Surgery, University Hospitals Cleveland Medical Center/Case Western Reserve University.

## Abstract

Emphysematous osteomyelitis (EO) is a rare condition identified through the presence of intraosseous gas. It is frequently fatal even with prompt recognition and management. We report a case of EO presenting with a necrotizing soft tissue infection of the thigh in the setting of prior pelvic radiation. The purpose of this study was to highlight the unusual association between EO and necrotizing soft tissue infection.

Emphysematous osteomyelitis (EO) is a rare osseous infection caused by gas-forming bacteria. Since it was first described in 1981 by Ram et al,^1^ only 57 cases have been reported in the literature. We report a patient diagnosed with EO of the pelvis associated with contiguous necrotizing soft tissue infection of the thigh.

## Case Report

An 88-year-old man with a history of urinary retention and recurrent urinary tract infection due to urethral stricture after radiation for prostatic adenocarcinoma presented to the emergency department with 1 day history of rapidly progressing left thigh erythema and swelling. He reported mild pain in his pelvis, hip, and knee. He had reported no history of constitutional symptoms.

**Figure 1:**
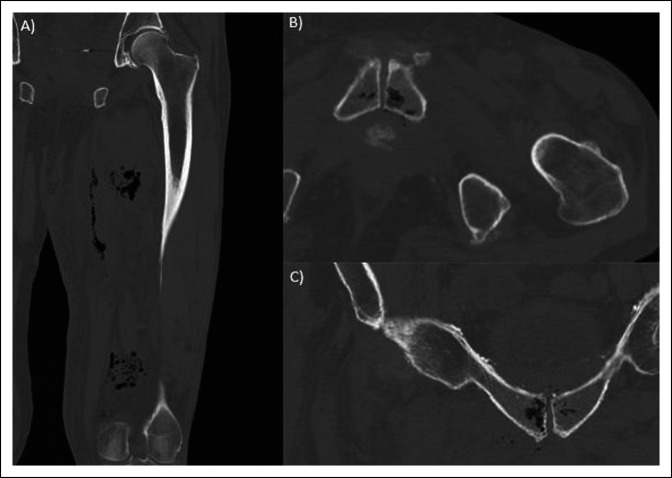
Computed tomography images from initial presentation. **A**, Coronal images of the thigh showing multiple foci of gas in the adductor compartment indicative of a necrotizing soft tissue infection. **B**, Axial and **C**, coronal images from pelvic CT showing intraosseous gas within the parasymphyseal bilateral superior pubic ramus.

The patient was afebrile and hemodynamically stable on presentation. A large area of erythema, swelling, and induration was observed with subcutaneous crepitus over the medial thigh concerning for necrotizing soft tissue infection. The white blood cell count was 9.7 × 10^9^ cells/L. Other notable laboratory results included sodium of 125 mmol/L, hemoglobin of 11.1 g/dL, glucose of 183 mg/dL, bicarbonate of 18 mmol/L, and lactate of 2.6 mmol/L. A CT scan of the left lower extremity showed extensive gas and fat stranding within the adductor compartment of the thigh (Figure 1). Gas was also seen in the adjacent contralateral pubic symphysis, consistent with EO. The patient was started on vancomycin, piperacillin/tazobactam, and clindamycin and was taken emergently for débridement of the left medial thigh.

On medial compartment fascial release, cloudy dish-water fluid was expressed. Intraoperative cultures were taken before thorough irrigation and excisional débridement. The posterior compartment of the thigh was explored, and frank purulent discharge was found to track up to the ipsilateral inferior pubic ramus. Bony débridement was initially deferred to better characterize the osseous extent of the infection through magnetic resonance imaging (MRI).

**Figure 2:**
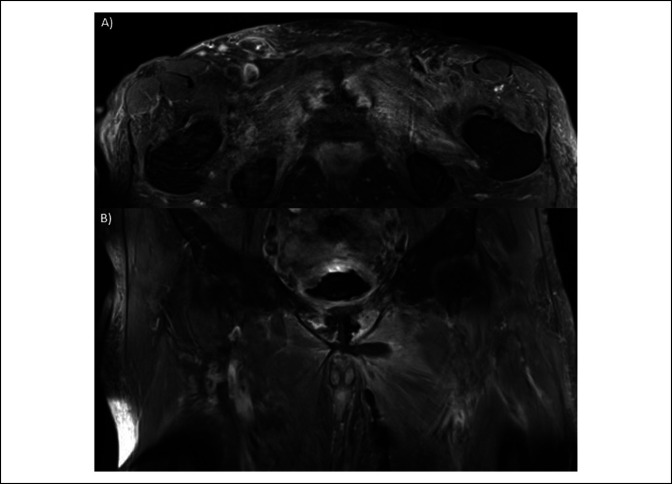
Magnetic resonance imaging of the pelvis showing hyperintense, rim-enhancing lesions along with hypointense pockets of gas on T2 postcontrast imaging. These areas of gas correspond to the areas seen on pelvic CT and are indicative of pubic bone emphysematous osteomyelitis.

MRI of the pelvis with and without gadolinium contrast on postoperative day 1 after the index débridement demonstrated diffuse edema on T2 sequences as well as rim enhancement and hypointense signal changes in the parasymphyseal pubis and inferior pubic rami bilaterally. Areas of hypointensity on MRI correlated with intraosseous gas seen on CT (Figure 2). The patient subsequently underwent a thorough irrigation and débridement of the pubic symphysis and repeat débridement of the thigh on hospital day 2. A Pfannenstiel incision was used to approach the symphysis, and the areas of osteomyelitis were débrided with a large rongeur and drill decompression. No purulent fluid was encountered during pubic débridement, but cultures of bone and surrounding soft tissue were collected. Vancomycin and tobramycin beads were placed into the space of Retzius and embedded into the parasymphyseal bones.

The patient recovered uneventfully in the intensive care unit and was transferred to a regular nursing floor on postoperative day 0 after pelvic irrigation and débridement. He remained hemodynamically stable throughout his admission. He was continued on broad spectrum antibiotics until cultures resulted. Cultures from the medial compartment of the thigh yielded mixed anaerobic species, while the bacterial cultures from the pubis grew mixed bacteria with a primary isolate of *Clostridium ramosum*. During admission, the patient underwent colonoscopy which showed multiple diverticula.

The patient was discharged in a stable condition to a skilled nursing facility on postoperative day 8 with amoxicillin-clavulanate. Antibiotic duration was for 6 months total. The patient returned for a repeat CT scan 3 weeks postoperatively showing postoperative fluid collections but no evidence of continued infection and remained infection-free at 8 weeks postoperatively.

## Discussion

EO is a rare condition that is commonly fatal. Patients present either with pain at the site of infection or with fever and constitutional signs of infection.^2^ Diagnosis requires a CT scan with evidence of intraosseous air, which is virtually pathognomonic if found outside of the vertebrae. Other rare causes of intraosseous air must be ruled out, including trauma, osteonecrosis, postoperative changes, neoplasm, or degenerative disease.^3^ MRI can be used to confirm the diagnosis and will show heterogenous signal hyperintensity in the areas of infection.

To our knowledge, only 57 cases of EO have been reported in the literature. Most of these cases (32) were reported after 2012, indicating that this condition may be underreported. The median age at diagnosis is 57 years (range 14 to 83). Our patient is the oldest patient to date reported with EO. Most patients with EO are immunocompromised. Of all case reports, 27 (47.4%) presented in the setting of uncontrolled diabetes, 8 (14%) had current or recently treated neoplasm, and 7 (12.3%) had a recent operation. Only eight patients (14%) had no immunocompromising factors. Uncontrolled diabetes is also associated with increased mortality from EO as well.^4,5^ Our patient had undergone radiation therapy for prostatic carcinoma, which has been reported in one prior case of EO.^6^ That patient similarly developed parasymphyseal EO thought to be caused by bowel or urinary fistula.^6^

EO most commonly presents in the vertebrae (30 cases, 52.6%) and pelvic bones/hips (21 cases, 36.8%) usually because of hematologic seeding. Nine cases (15.7%) were multifocal. Three prior cases reported by Merine et al developed after contiguous extension from abdominal/pelvic abscesses, while three cases developed in the setting of chronic urinary tract infection/pyelonephritis.^4,7-9^ Only one patient has developed EO in the setting of necrotizing fasciitis.^5^ This patient had EO of the right midfoot and underwent an urgent below knee amputation.

Necrotizing soft tissue infection after radiation therapy for uropelvic carcinoma has been reported in the literature twice before our case.^10,11^ Both patients developed uropelvic fistulas that were thought to be the source of necrotizing soft tissue infection, although EO was not found in either case. Both patients recovered after surgical débridement and a long course of antibiotics.^10,11^ Necrotizing soft tissue infection has also been reported after radiation therapy for rectal cancer.^12,13^ Given the similarities between these cases and ours, it appears that pelvic radiation may serve as a risk factor for severe bone and soft tissue infections.

The bacterial species commonly found in cultures of EO are members of the Enterobacteriaceae family. Monomicrobial infection is much more common in EO because only 12 of the reported cases were polymicrobial (21%). Causative organisms for EO include *Escherichia coli* (15 cases, 26.3%), *Klebsiella pneumoniae* (10 cases, 17.5%), *Fusobacterium necrophorum* (4 cases, 7%), and various *Salmonella* species (3 cases, 5.3%). Only one prior reported case was positive for *Clostridium* species on culture,^14^ and no prior cases have isolated *Clostridium ramosum* on bone culture.

*Clostridium ramosum* a commensal intestinal organism in humans. It usually is found as a component of polymicrobial soft tissue infections but is rarely found as an isolated cause of infection due to low virulence. However, given challenges with identification, it is likely underdiagnosed on culture.^15^ Very few cases have been reported of *Clostridium ramosum* as a cause for orthopaedic infections: one case of spondylodiscitis [Lavigne], one case of pseudarthrosis,^16^ one case of septic arthritis,^17^ and two cases of osteomyelitis.^18,19^ Our case is the first reported case of EO caused primarily by this rare pathogen. Given the prevalence of this bacterium in the gastrointestinal tract, our patient underwent a colonoscopy during admission, but no obvious source of infection was discovered. Possible sources of infection included undiagnosed or contained diverticular perforation versus seeding from prior prostatic procedures.

Given the rarity of EO, there are no available guidelines for treatment. Our patient was started on broad-spectrum antibiotics to ensure sufficient bacterial coverage before surgical management. Of the reported cases, 63% required at least one surgery. Surgical débridement seems to be related to survival because 6 of the 14 patients (43%) who expired before discharge were treated surgically, while 24 of the 32 patients (66.7%) who recovered had surgical management. Intramedullary antibiotics were used in one prior case, which also resulted in successful eradication of infection.^20^ The overall mortality rate in cases with reported outcomes was 30.4% (14/46 cases), indicating the significant morbidity of this condition irrespective of medical and surgical management.

We report a rare case of EO of the pubis in the setting of prior pelvic radiation, urethral stricture, and concomitant necrotizing soft tissue infection of the left thigh. In addition, this is the first reported case of EO with *Clostridium ramosum* as the primary causative organism. Regardless of the setting, after detection of EO through intraosseous air on the CT scan, early and aggressive surgical débridement and broad antibiotic therapy are essential for the treatment of this condition.
